# A Decade of Data from an IVF Center: Factors Contributing to Infertility, Their Prevalence, and Impact on Live Birth Rates

**DOI:** 10.3390/jcm15176609

**Published:** 2026-08-27

**Authors:** Hale Bayram, Yaprak Dönmez Çakıl, Belgin Selam, Mehmet Cıncık

**Affiliations:** 1 Department of Histology and Embryology, Faculty of Medicine, Maltepe University, 34857 İstanbul, Türkiye; halebayram@maltepe.edu.tr (H.B.); mehmet.cincik@maltepe.edu.tr (M.C.); 2 Department of Medical Biology and Genetics, Faculty of Medicine, Maltepe University, 34857 İstanbul, Türkiye; yaprak.cakil@maltepe.edu.tr; 3 Department of Obstetrics and Gynecology, School of Medicine, Acibadem Mehmet Ali Aydinlar University, 34752 İstanbul, Türkiye

**Keywords:** in vitro fertilization, assisted reproductive technology, intracytoplasmic sperm injection, male infertility, female infertility, unexplained infertility, pregnancy, live birth

## Abstract

**Background:** Infertility is increasingly recognized as a significant global public health concern. A thorough understanding of the diverse causes of infertility and their influence on treatment outcomes is essential for optimizing therapeutic strategies and improving success rates. **Methods:** In this study, we analyzed data from 9132 couples who underwent IVF treatment at a fertility center in Istanbul, Turkey, between 2010 and 2020. **Results:** Female factor infertility was the most prevalent among all participants (35.4%), followed by male infertility (26.4%), combined infertility (22.3%), and unexplained infertility (15.8%). Diminished ovarian reserve was found to be the most common indication in the female and combined infertility groups. Embryo transfer (ET) was performed in 7485 cases. Within this cohort, the antral follicle count was higher in the combined and unexplained infertility groups compared with the female and male infertility groups. The highest number of MII oocytes was found in the unexplained infertility group. Pregnancy rates varied significantly across groups, being higher in the male infertility and unexplained infertility groups than in the female infertility and combined infertility groups. Live birth rates also varied significantly across groups. The highest rate was observed in the unexplained infertility group. **Conclusions:** The distribution of infertility etiologies and their association with ICSI outcomes underscore the necessity to enhance the efficacy of ART protocols and to adopt evidence-based approaches to treatment strategies. Female factor infertility remains a significant clinical challenge, whereas unexplained infertility presents relatively favorable outcomes.

## 1. Introduction

Infertility is increasingly recognized as a significant global public health concern. A growing body of evidence documents its rising prevalence across diverse populations [1]. Parallel to this trend is a substantial increase in the use of assisted reproductive technologies (ART), particularly in vitro fertilization (IVF). National survey data from Turkey indicates that the proportion of individuals undergoing IVF treatment rose from 1.9% in 2008 to 4.1% in 2013 [2].

Infertility is frequently a multifactorial condition, involving both female and male factors [3]. This issue can be attributed to a combination of biological, genetic, environmental, and lifestyle factors [4,5]. Historically, infertility research has predominantly focused on female reproductive health due to the complexity of its etiology. However, there has been a recent increase in attention to male infertility primarily due to growing evidence of its significant contribution to overall infertility rates and advancements in diagnostic technologies [5,6].

The etiology of female infertility comprises a wide spectrum of possible etiologies, including, but not limited to, ovulatory dysfunction, fallopian tube obstruction, endometriosis, advanced maternal age, and uterine abnormalities. Ovulatory disorders, often linked to conditions such as polyendocrine metabolic ovarian syndrome (PMOS), can result in irregular or absent ovulation, significantly impairing fertility [4,7]. Fallopian tube obstructions can prevent fertilization by hindering the convergence of the oocyte and sperm, thereby reducing the likelihood of conception. Endometriosis, defined as the abnormal growth of endometrial tissue outside the uterus, can impair reproductive function by affecting the fallopian tubes, ovaries, and uterine environment. The presence of endometriotic lesions or ovarian endometriomas was considered a significant factor contributing to infertility, particularly when associated with pelvic adhesions or distorted ovarian anatomy [8]. Furthermore, advanced maternal age is strongly correlated with diminished ovarian reserve (DOR) and declined oocyte quality, leading to increased rates of infertility in women over 35 years [9]. Congenital or acquired uterine abnormalities have also been demonstrated to interfere with implantation or increase the risk of miscarriage [10]. Additional contributing factors may include cervical abnormalities and immunological or genetic disorders, although these are less common [11].

Male infertility has been identified as a significant underlying cause of reproductive complications, commonly resulting from low sperm count (oligozoospermia), impaired sperm motility (asthenozoospermia), and abnormal sperm morphology (teratozoospermia). Underlying causes often include varicocele, hormonal imbalances, genetic abnormalities, and, in certain instances, environmental or lifestyle factors such as smoking, obesity, and exposure to toxins [5,6]. In many cases, infertility arises from a combination of male and female factors, necessitating a couple-centered approach to diagnosis and treatment [12].

While chromosomal imbalances and genetic anomalies are not direct causes of infertility, they can interfere with normal embryo development, leading to implantation failure and recurrent pregnancy loss [13]. Preimplantation genetic diagnosis (PGD), a prenatal diagnostic procedure conducted on early-stage embryos created through IVF, enables the screening and transfer of euploid embryos, thereby improving clinical outcomes [14,15]. This technique has been shown to be particularly beneficial for patients with a high risk of aneuploidy, including those with recurrent miscarriage, advanced maternal age, unexplained infertility, or consistently poor embryo quality [16,17].

Although infertility is increasingly prevalent and significant progress has been made in ART, a thorough understanding of the factors influencing treatment outcomes remains incomplete. This study aims to conduct a comprehensive evaluation of both female and male infertility factors, their changes over the years, and their association with live birth outcomes, utilizing a decade of clinical data. By examining these factors in detail, this research aims to contribute to the optimization of ART protocols and the implementation of evidence-based approaches in infertility management.

## 2. Materials and Methods

### 2.1. Participants

This retrospective study examined the infertile couples who underwent ICSI treatment at the Acıbadem Altunizade IVF Center in Istanbul between 2010 and 2020. A total of 9132 couples were included, including both those whose post-embryo transfer follow-up was conducted at this center and those who underwent follow-up at external centers. The cases included female infertility, male infertility, combined (female and male) infertility, and unexplained infertility. The study also included couples undergoing PGD-ICSI treatments due to a history of aneuploid pregnancy. Couples whose medical records could not be accessed and those who did not consent to the use of their data for research purposes were excluded from the study. Only one ICSI cycle per couple was included in the analysis to avoid duplication and ensure independent observations. The study protocol was approved by the Maltepe University Non-Interventional Scientific Research Ethics Committee (approval date: 20 February 2025; approval number: 2025/04-04).

### 2.2. Diagnosis and Assessment of Infertility

The evaluation of tubal factors in female infertility was performed using several diagnostic methods, with hysterosalpingography (HSG) being the most commonly used technique to assess tubal patency and the structural integrity of the fallopian tubes, along with laparoscopy combined with chromopertubation [18,19]. Ovulatory dysfunction was assessed through a combination of patient history, physical examination, and laboratory tests, including measurements of follicle-stimulating hormone (FSH), luteinizing hormone (LH), thyroid-stimulating hormone (TSH), and prolactin levels. Additionally, antral follicle count (AFC) was assessed by transvaginal ultrasound. In cases of suspected PMOS, additional evaluations included measurements of free and total testosterone, dehydroepiandrosterone sulfate (DHEAS), and 17-hydroxyprogesterone (17-OHP) [20]. DOR was defined according to the Bologna criteria, requiring at least two of the following: advanced maternal age (≥40 years) or other risk factors, a previous poor ovarian response, and abnormal ovarian reserve tests. Serum anti-Müllerian hormone (cut-off values 0.5–1.1 ng/mL), estradiol levels (≥80 pg/mL), and AFC (<5) were measured as part of the evaluation [21,22]. The initial evaluation of endometriosis and endometrioma primarily involved transvaginal ultrasound, with confirmation by laparoscopy when indicated [23].

Semen samples from male partners were collected after three to five days of sexual abstinence and analyzed in the same laboratory under standardized conditions. For the present study, all samples were re-evaluated in accordance with the World Health Organization (WHO) 2021 criteria. Sperm classification was performed based on concentration and motility parameters. Participants were classified as normozoospermic (16 × 10^6^/mL and above), oligozoospermic (below 16 × 10^6^/mL), or azoospermic (no sperm in the ejaculate) based on sperm concentration. Men with ejaculates showing less than 30% progressively motile spermatozoa were categorized as asthenozoospermic, and those with a percentage of sperm with normal morphology below 4% were diagnosed with teratozoospermia [24]. The micro-TESE method was used for men diagnosed with azoospermia as previously described [25].

### 2.3. ICSI-Based Fertilization and Embryo Transfer

Spermatozoa obtained through masturbation or micro-TESE were injected into mature oocytes using the micromanipulation technique. Fertilization was assessed by the identification of two pronuclei and two polar bodies 12–18 h after the ICSI procedure [26,27]. As a substantial body of literature has demonstrated no statistically significant difference in pregnancy and live birth outcomes between fresh and frozen–thawed embryo transfer (ET) [28], all cases undergoing ET were included in this study.

ET was performed on day 3 or 5 based on the quality of the embryo and the patient’s clinical profile, in accordance with the standard practice guidelines of the European Society of Human Reproduction and Embryology (ESHRE). According to the transfer policies set by the Ministry of Health of the Republic of Türkiye, women under the age of 35 are permitted to undergo a single ET, while women aged 35 and older, or those under 35 with two or more previous failed ART attempts, may undergo the transfer of up to two embryos [29].

### 2.4. Pregnancy Assessment

Of the 7485 cases that underwent ET, serum β-human chorionic gonadotropin (β-HCG) levels were assessed 10–12 days after ET. Pregnancy was considered positive if the serum β-HCG level was greater than 10 mIU/mL. In β-HCG-positive cases, the gestational sac was examined by transvaginal ultrasound at four to six weeks.

### 2.5. Statistical Analysis

Statistical analyses were performed using the Statistical Package for the Social Sciences (SPSS) version 26.0 (IBM SPSS Statistics, Armonk, NY, USA). Quantitative variables were presented as mean and standard deviation, and qualitative variables were summarized using descriptive statistics, such as frequency and percentage. The Shapiro–Wilk test and box plots were used to assess the normality of the data distribution. One-way ANOVA, followed by Tukey’s post hoc test, was used to compare normally distributed quantitative variables across three or more groups. The Pearson chi-squared test with Bonferroni correction was used to compare categorical variables. A 95% confidence interval was used, and *p*-values less than 0.05 were considered statistically significant.

## 3. Results

Table 1 presents the descriptive characteristics of the couples included in the study. The mean maternal age of the cases included in the study was identified as 34.84 ± 5.69 years, while the mean paternal age was found to be 37.44 ± 6.24 years. The mean body mass index (BMI) of the 8536 women for whom height and weight data were available was calculated as 23.94 ± 4.05 kg/m^2^. When smoking status was evaluated, it was found that 68.60% of the women did not smoke, 26.80% smoked fewer than 20 cigarettes per day, and 4.60% smoked more than 20 cigarettes per day. During the study period, 1913 (20.90%) of the couples who sought IVF treatment were recorded between 2010 and 2013, 4126 (45.20%) between 2014 and 2017, and 3093 (33.90%) were recorded between 2018 and 2020. Among the 3238 β-hCG-positive cases, the live birth rate was 50.80% (*n* = 1644). A chemical pregnancy was observed in 25.40% of cases (*n* = 821); an abortion followed in 14.60% (*n* = 474). Ectopic pregnancy was relatively rare, accounting for 2.60% of cases (*n* = 84). Data on the pregnancy outcome were not available in 6.6% of cases (*n* = 215).

**Figure 1:**
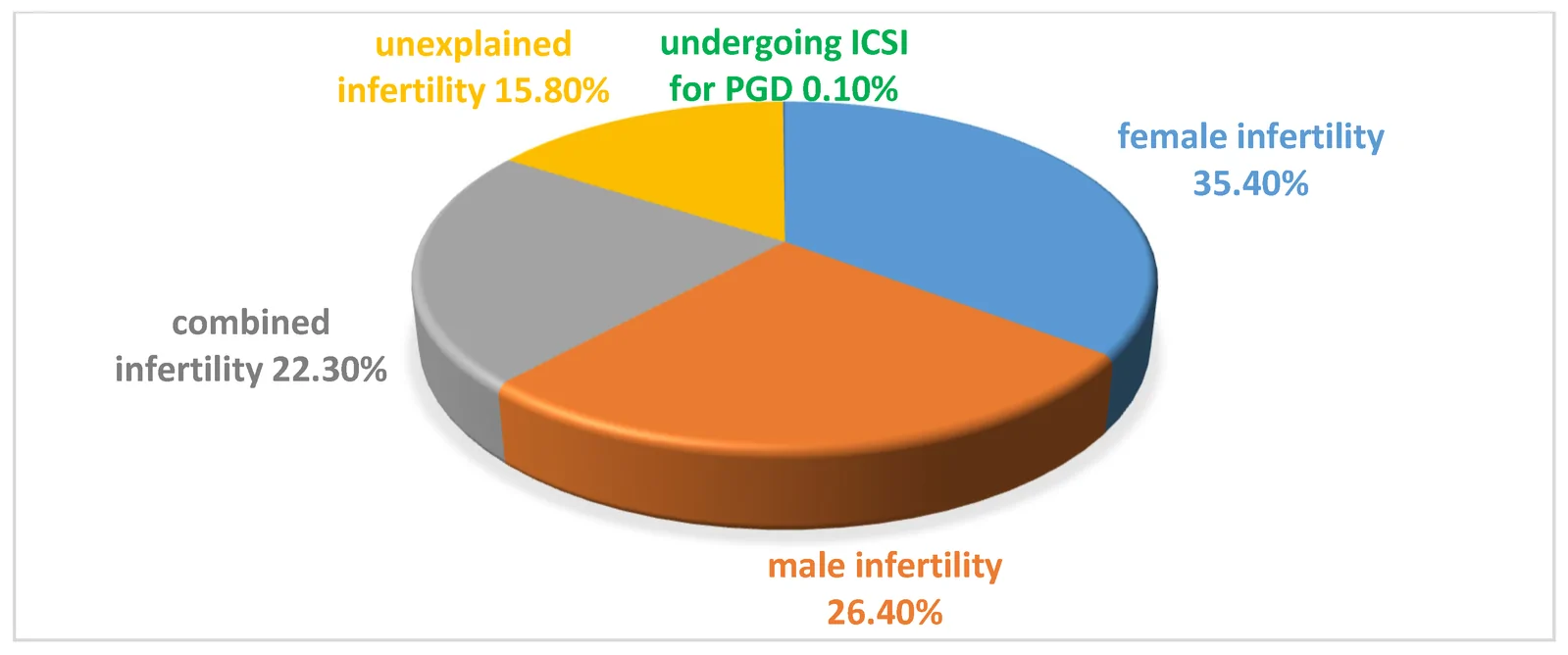
Distribution of indications for ICSI treatment. The data of the 9132 couples who underwent ICSI treatment over the 10-year study period are shown.

Figure 1 displays the causes of infertility among the 9132 couples who underwent ICSI treatment over the 10-year study period. Accordingly, the ART indications of the cases were reported as female infertility in 35.4% (*n* = 3235), male infertility in 26.4% (*n* = 2409), combined infertility in 22.3% (*n* = 2033), and unexplained infertility in 15.8% (*n* = 1444) of the cases. The number of cases undergoing ICSI for PGD was 11 (0.1%).

The cases included in the study were divided into three periods based on the years of the study: 2010–2013 (x), 2014–2017 (y), and 2018–2020 (z), and the distribution of maternal age, paternal age, etiologies of infertility, cases undergoing ET, and IVF outcomes was compared in Table 2. Maternal age increased significantly over the years (*p* < 0.001). The mean maternal age was 33.65 ± 5.66 years during the 2010–2013 period, while it was significantly higher during the 2014–2017 (35.06 ± 5.70 years) and 2018–2020 (35.29 ± 5.60 years) periods. No difference was observed between the last two periods. Similarly, the paternal age also demonstrated a significant increase over the years (*p* < 0.001). The mean paternal age was 36.25 ± 6.13 years during the 2010–2013 period and was significantly higher during the 2014–2017 (37.72 ± 6.39 years) and 2018–2020 (37.81 ± 6.04 years) periods.

When the etiologies of infertility were examined, the female infertility rate was significantly higher in 2014–2017 (37.30%) than in both 2010–2013 (34.90%) and 2018–2020 (33.30%) (*p* < 0.01). The rate of male-factor infertility, however, was found to be lower during the 2014–2017 period (24.30%) compared to the other two periods (*p* < 0.001). The rate of combined infertility showed a gradual increase over the years, with rates of 19.70%, 22.60%, and 23.40%, respectively (*p* < 0.01). The unexplained infertility rate showed a significant decrease over the years (*p* < 0.001). The highest rate was observed in 2010–2013 (18.20%), followed by 2014–2017 (15.70%), with the lowest rate observed in 2018–2020 (14.50%). The rate of cases undergoing embryo transfer (ET) has decreased significantly over the years (*p* < 0.001). While the rate of cases undergoing ET was 92.10% during the 2010–2013 period, this rate declined to 81.70% during the 2014–2017 period and to 76.00% during the 2018–2020 period.

The β-hCG positivity rate, on the other hand, showed no significant changes over the years (*p* > 0.05). The β-hCG positivity rate, which was 44.69% during the 2010–2013 period, was found to be 43.58% and 41.73%, respectively, during the 2014–2017 and 2018–2020 periods. Among β-hCG-positive patients, live birth rates were found to be 49.9%, 51.3%, and 50.7%, respectively, across the study periods, and no significant difference was detected among the groups (*p* > 0.05). Also, no statistically significant differences were observed among the study periods in chemical pregnancy rates (25.4%, 26.6%, and 23.4%) or abortion rates (14.7%, 15.0%, and 14.1%) (*p* > 0.05). Ectopic pregnancy rates (2.2%, 2.1%, and 3.7%) increased in the 2018–2020 period (*p* < 0.01).

In all infertility cases, AFC ranged from 0 to 28, with an average of 10.44 ± 7.29, while the number of metaphase II (MII) oocytes ranged from 0 to 18, with an average of 4.96 ± 3.92.

Table 3 displays the distribution of etiologies of female, male, and combined infertility (*n* = 7677), excluding 1444 unexplained infertility cases whose etiology was unknown and 11 PGD cases. DOR was the most common indication (*n* = 1410, 43.6%) among female infertility cases (*n* = 3235). Subsequent to this, 17.2% (*n* = 556) of the cases exhibited PMOS, 10.5% (*n* = 341) displayed tubal factor infertility, 8% (*n* = 260) exhibited endometriosis, and 7.5% (*n* = 241) exhibited endometrioma. Furthermore, the analysis revealed the presence of combined etiologies of female infertility, with DOR coexisting with other female factors in 5.6% (*n* = 94), PMOS coexisting with other female factors in 3.3% (*n* = 107), tubal factor coexisting with other female factors in 6.2% (*n* = 201), endometriosis coexisting with other female factors in 3.4% (*n* = 108), and endometrioma coexisting with other female factors in 2.1% (*n* = 71) of the cases. Additionally, other etiologies accounted for 122 cases (3.7%). These less frequently reported causes included oocyte cryopreservation (3.4%, *n* = 110), advanced maternal age (1.7%, *n* = 55), uterine myomas (1.5%, *n* = 47), anovulation (1.1%, *n* = 34), endometrial polyps (0.3%, *n* = 8), and adenomyosis (0.1%, *n* = 2). There were isolated cases of cervical insufficiency, breast cancer, and vaginismus, each observed in one patient (*n* = 1).

Among male infertility cases, the most prevalent factor was oligozoospermia, observed in 56.0% of the cases (*n* = 1349), followed by azoospermia in 24.4% (*n* = 589), and asthenozoospermia in 19.6% (*n* = 471).

In the combined infertility group (*n* = 2033), etiologies involving DOR were the most prevalent. The most frequently observed combination was DOR with abnormal sperm parameters, including DOR with oligozoospermia, accounting for 29.3% of cases (*n* = 596); DOR with asthenozoospermia, observed in 10.7% of cases (*n* = 217); and DOR with azoospermia, observed in 4.9% of cases (*n* = 101). The second most frequent combined etiology was PMOS with abnormal sperm parameters. This included PMOS with oligozoospermia, observed in 18.8% (*n* = 386) of cases, followed by PMOS with azoospermia, observed in 6.2% (*n* = 127) of cases; and PMOS with asthenozoospermia, observed in 5.9% (*n* = 120) of cases.

Endometriosis/endometrioma combined with male factor abnormalities was also reported, though less frequently. Endometriosis/endometrioma combined with azoospermia was observed in 9.0% (*n* = 182) of cases, with asthenozoospermia in 2.8% (*n* = 57), and with oligozoospermia in 0.6% (*n* = 12). Similarly, among cases of tubal factor infertility combined with male factors, tubal factor infertility combined with azoospermia was reported in 7.5% (*n* = 150), asthenozoospermia in 3.2% of cases (*n* = 65), and oligozoospermia in 0.2% of cases (*n* = 4).

Less frequently observed combinations included anovulation with male factor infertility (0.8%, *n* = 3), uterine myomas with male factor infertility (1.4%, *n* = 5), endometrial polyps with male factor infertility (0.6%, *n* = 2), and advanced maternal age with male factor infertility (1.1%, *n* = 4, Table 3).

ET was performed in 7485 cases, with single ET in 4981 cases and double ET in 2504 cases. ET could not be performed in 1647 cases due to various factors, including failed gamete retrieval, poor embryo development, insufficient endometrial thickness, or the presence of ovarian hyperstimulation syndrome. As presented in Table 4, female factor infertility was the most prevalent (33%), followed by male factor infertility (26.7%), combined infertility (22.2%), and unexplained infertility (18%).

The mean maternal age was significantly higher in the female and combined infertility groups than in the male and unexplained infertility groups (*p* < 0.001 for all). However, there was no difference between the female infertility and combined infertility groups (*p* > 0.05). A statistically significant difference in mean paternal age was observed among the infertility groups (*p* < 0.001). Paternal age was highest in the combined infertility group. Compared to the female infertility group, the mean paternal age was significantly lower in the unexplained infertility group (*p* < 0.001). At the same time, there was no difference between the female infertility and male infertility groups (*p* > 0.05). Similarly, there was no difference between the male infertility and unexplained infertility groups (*p* > 0.05).

A significant difference in mean AFC was found among the groups (*p* < 0.001). Mean AFC was higher in the combined infertility and unexplained infertility groups in comparison to the female infertility and male infertility groups (*p* < 0.001 for all comparisons). There was no significant difference in mean AFC between the female infertility and male infertility groups, and between the combined infertility and unexplained infertility groups (*p* > 0.05 for both).

Similarly, the mean number of MII oocytes varied significantly among the groups (*p* < 0.001). The largest quantity of MII oocytes was retrieved from the unexplained infertility group. This group had a significantly higher number of MII oocytes than the female infertility, male infertility, and combined infertility groups (*p* < 0.001). The lowest number of MII oocytes was collected from the female infertility group (Table 4).

A statistically significant difference was recorded in pregnancy rates among the infertility groups (*p* < 0.01). The male infertility and unexplained infertility groups showed significantly higher pregnancy rates compared to both the female and the combined infertility groups (*p* < 0.01 for all comparisons).

Live birth rates in cases undergoing ET also showed a statistically significant difference among groups (*p* < 0.01). The unexplained infertility group had the highest live birth rate compared to the other groups (*p* < 0.01 for all comparisons). In addition, when compared with the male infertility group, the female infertility group and the combined infertility group exhibited significantly lower live birth rates (*p* < 0.01 for all comparisons). Similar findings were recorded for live birth rates in β-HCG-positive cases (Table 4). The ectopic pregnancy rates in the female, male, combined, and unexplained groups were found to be 5.3%, 2.9%, 5.1%, and 2.4%, respectively, and no statistically significant difference was observed between the groups (*p* > 0.05). Chemical pregnancy rates ranged from 26.7%, 24.4%, 28.4%, and 19.8%, respectively. The difference between the female, combined, and unexplained groups was statistically significant (*p* < 0.01). Abortion percentages were 14.6%, 14.2%, 13.7%, and 12.5% in the female, male, combined, and unexplained groups, respectively, and no significant difference was found among the groups (*p* > 0.05). Similarly, not available rates were 6.4%, 7.9%, 4.4%, and 5.6%, respectively, and no statistically significant difference was found between the groups (*p* > 0.05).

## 4. Discussion

Infertility is a common health issue worldwide, with its prevalence steadily rising in recent years [1]. Despite substantial advances in ART, many couples still do not achieve successful outcomes. A growing body of evidence highlights the crucial influence of underlying infertility etiologies on ART success, with direct implications for pregnancy and live birth rates. In this study, we evaluated infertility factors and their association with pregnancy and live birth outcomes over a ten-year period at an IVF center. Given that the ultimate measure of ART success is the achievement of live birth, it is essential to investigate the factors that most significantly influence this critical endpoint. This retrospective study provides comprehensive insights into the distribution of infertility causes among couples undergoing ICSI treatment, while comparing pregnancy and live birth rates across female factor, male factor, and unexplained infertility. In addition, the impact of female and male age on reproductive outcomes was evaluated. To our knowledge, this study represents the largest cohort reported from Türkiye to date.

In this study, the overall cohort consisted of 9132 couples. Maternal mean age was 34.84 ± 5.69 years, while paternal mean age was 37.44 ± 6.24 years. The mean BMI was 23.94 ± 4.05 kg/m^2^, and the majority of the study population was within the normal BMI range. The majority of women were non-smokers (68.6%); 26.8% reported smoking fewer than 20 cigarettes per day, while 4.6% reported smoking 20 or more cigarettes per day. Although the highest number of IVF treatment requests was recorded between 2014 and 2017, it should be noted that the 2018–2020 period was one year shorter than the other study periods. When this is taken into account, the findings indicate an increase in the rate of IVF treatments over the years [2]. Live births are the predominant outcome among β-hCG-positive cases, which is a favorable outcome.

Comparisons across study periods indicate changes over time in maternal and paternal ages, infertility etiology, and IVF outcomes. The maternal and paternal ages of individuals seeking IVF treatment increased over time. This finding is consistent with studies reporting a global trend toward older age among individuals seeking infertility treatment. Prolonged education, career planning, socioeconomic factors, and increased access to ART may have led couples to postpone having children until later in life. Advanced maternal age has been reported to negatively affect oocyte quality and embryo development potential, while advanced paternal age may influence sperm DNA integrity and embryo development [30,31].

When the causes of infertility were evaluated, the gradual increase in combined infertility was notable. This trend may be related to the fact that, as a result of more detailed evaluation of both partners and advances in diagnostic approaches during the assessment of infertile couples, multiple factors can now be identified simultaneously, rather than a single cause [32]. Furthermore, a decrease in unexplained infertility rates may be attributed to advances in diagnostic methods for identifying the causes of infertility, as well as the more widespread use of ovarian reserve assessment, genetic testing, advanced sperm evaluation methods, and imaging techniques [33]. Currently, increased awareness of male infertility and the more widespread use of diagnostic evaluations may have contributed to the more accurate identification of cases in which male factors play a role [34].

During the 2014–2017 and 2018–2020 periods, there was a decrease in the ET rates performed. This decrease observed in subsequent years may be partly attributed to the increase in maternal age within the study population. Several studies have reported that as maternal age increases, cancelation of an IVF cycle due to poor ovarian response or the lack of transferable embryos also becomes more likely. Consequently, decreases in embryo transfer rates may be associated with increasing maternal age [35]. In addition, during the pandemic, in accordance with the recommendations of international reproductive medicine associations, treatment cycles were delayed, elective embryo transfers were temporarily suspended, or embryos were frozen, and transfers were scheduled for a later date [36]. Therefore, the ET rate may also have decreased.

Among cases undergoing ET, the β-hCG positivity rate did not differ significantly across the study periods. Moreover, no significant differences were observed over time in live birth, chemical pregnancy, or abortion rates among cases that were β-hCG-positive. Importantly, the fact that β-hCG positivity rates remained comparable suggests favorable embryo transfer outcomes throughout the study period. Ectopic pregnancy rates were found to be high between 2018 and 2020. Increased maternal and paternal ages may be the reason for this. It is well known that advanced maternal age has adverse effects on obstetric complications [30,31].

The prevalence of female infertility was found to be higher than that of other types of infertility, a finding consistent with broader evidence in the literature. Jabeen et al. [37], in their systematic review on primary and secondary infertility, noted female infertility as the most common category, and Barbieri [38] similarly described female factors as the predominant cause. Notably, in the UK, Bhattacharya et al. reported that approximately 50% of infertility cases were attributable to female factors. In the same study, the prevalence of unexplained infertility has been reported to range from 10% to 20% [39]. Nonetheless, some studies have indicated that both male and female factors contribute equally to infertility [40,41].

Female infertility is a complex health problem that affects many women of reproductive age and can develop due to various factors. The frequency and impact of these factors may vary depending on individual characteristics and social factors. In the present study, DOR was identified as the most prevalent cause of female infertility, followed by PMOS, tubal factors, and endometriosis/endometrioma, consistent with previous studies [42,43,44,45,46].

The assessment of male fertility is largely dependent on the evaluation of semen quality [47,48]. Milardi et al. and Pant et al. have shown that abnormalities in sperm count are the most common abnormalities and are generally the most significant factor contributing to male infertility [49,50]. In this study, the most common cause of male infertility was identified as oligozoospermia, followed by azoospermia, with asthenozoospermia being the least frequently observed condition. Similarly, in another study evaluating semen analysis reports of 3084 infertile couples in India, the highest rate was observed for oligozoospermia, a finding that aligns with the results of this study. This was followed by asthenozoospermia, while azoospermia was observed at the lowest rate [51].

The higher mean maternal age observed in the female infertility group in our study can be attributed to the well-established impact of age on female reproductive health. As women age, particularly after the age of 35, ovarian reserve declines, resulting in a decrease in both the quantity and quality of oocytes. This decline in reproductive potential, combined with an increased risk of age-related conditions such as ovulatory dysfunction and tubal blockages, likely contributes to the greater prevalence of infertility among older women [52,53]. Similarly, the elevated mean paternal age observed in the combined infertility group can be attributed to unfavorable effects of advanced age on sperm concentration, progressive motility, and live births, particularly evident after the age of 40 [31]. This could explain the higher prevalence of infertility in couples with both male and female factors, as both partners may synergistically impair fertility potential with advancing age [54,55].

The present study revealed significant differences in AFC and the number of MII oocytes among groups with varying infertility etiologies. As expected, the female infertility group exhibited the lowest mean AFC and MII oocyte numbers compared to the other groups. This finding is largely attributable to the higher prevalence of DOR within this subgroup. DOR is the leading cause of female infertility and can affect women across a wide age spectrum [56]. While more prevalent among older women, recent studies have reported an increasing number of diagnoses among younger women [45].

Only a paucity of studies exists that directly compare pregnancy outcomes across groups stratified by female, male, combined, and unexplained infertility factors. In our study, the highest pregnancy and live birth rates were observed in the unexplained infertility group, followed by the male infertility group, whereas the lowest live birth rates were found in the female infertility group.

## 5. Conclusions

This retrospective study, which includes one of the largest sample sizes reported from Türkiye, highlights the significant impact of infertility factors on determining ART outcomes. Female infertility was the most prevalent category, with DOR emerging as the leading cause. The highest live birth rates were associated with unexplained infertility, followed by male infertility, while female infertility yielded the lowest success rates. This study provides valuable insights into the prevalence and impact of infertility factors, which can inform clinical decision-making to optimize ART treatment outcomes.

## Figures and Tables

**Table 1:** Participant descriptive characteristics.

		*n* = 9132
Maternal age (mean ± SD)		34.84 ± 5.69
Paternal age (mean ± SD)		37.44 ± 6.24
BMI (kg/m^2^) (*n* = 8536), (mean ± SD)		23.94 ± 4.05
Smoking status, *n* (%)	Non-smoker	6262 (68.60)
	<20 cigarettes/day	2450 (26.80)
	≥20 cigarettes/day	420 (4.60)
IVF application year, *n* (%)	2010–2013	1913 (20.90)
	2014–2017	4126 (45.20)
	2018–2020	3093 (33.90)
Pregnancy outcomes for β-HCG positive cases	Live birth, *n* (%)	1644 (50.80)
	Ectopic, *n* (%)	84 (2.60)
	Chemical, *n* (%)	821 (25.40)
	Abortion, *n* (%)	474 (14.60)
	Not available, *n* (%)	215 (6.60)

**Table 2:** The distribution of maternal age, paternal age, etiologies of infertility, cases undergoing ET, and IVF outcomes by study period among 9132 infertility cases.

		^x^ 2010–2013	^y^ 2014–2017	^z^ 2018–2020	*p*	Post Hoc
^a^ Maternal age		33.65 ± 5.66	35.06 ± 5.70	35.29 ± 5.60	*p* = 0.000 ***	x < y, z
^a^ Paternal age		36.25 ± 6.13	37.72 ± 6.39	37.81 ± 6.04	*p* = 0.000 ***	x < y, z
^b^ Female factor %		34.90	37.30	33.30	*p* = 0.004 **	x, z < y
^b^ Male factor %		27.00	24.30	28.80	*p* = 0.000 ***	y < x, z
^b^ Combined infertility%		19.70	22.60	23.40	*p* = 0.001 **	x < y < z
^b^ Unexplained infertility %		18.20	15.70	14.50	*p* = 0.000 ***	z < y < z
^b^ Cases undergoing ET %		92.10	81.70	76.00	*p* = 0.000 ***	z < y < z
^b^ β-HCG positive cases %		44.69	43.58	41.73	*p* = 0.145	y, z, x
^b^ Pregnancy outcomes in β-HCG positive cases (*n* = 3238)	Live birth %	49.90	51.30	50.70	*p* = 0669	x, y, z
	Ectopic (%)	2.20	2.10	3.70	*p* = 0.002 **	x, y y < z x < z
	Chemical (%)	25.40	26.60	23.40	*p* = 0.319	x, y, z
	Abortion (%)	14.70	15.00	14.10	*p* = 0.594	x, y, z
	Not available (%)	7.80	5.00	8.20	*p* = 0.001 **	x, y x, z y < z

^a^ one-way ANOVA. Post hoc pairwise comparisons were performed using Tukey. ^b^ Pearson’s chi-square test. Post hoc pairwise comparisons were performed using Bonferroni correction. ^x^ 2010–2013; ^y^ 2014–2017; ^z^ 2018–2020. Symbols (x, y, and z) indicate the groups compared in the post hoc analysis. ** *p* < 0.01, *** *p* < 0.001.

**Table 3:** The distribution of etiologies of female, male, and combined infertility in 7677 infertility cases.

Female İnfertility (*n* = 3235)		*n* (%)
	DOR	1410 (43.6)
	PMOS	556 (17.2)
	Tubal factor	341 (10.5)
	Endometriosis	260 (8)
	Endometrioma	241 (7.5)
	DOR + other female factor	94 (5.6)
	PMOS + other female factor	107 (3.3)
	Tubal + other female factor	201 (6.2)
	Endometriosis +other female factor	108 (3.4)
	Endometrioma + other female factor	71 (2.1)
	Other factors *	122 (3.7)
Male infertility (*n* = 2409)		
	Asthenozoospermia	471 (19.6)
	Azoospermia	589 (24.4)
	Oligozoospermia	1349 (56)
Combined infertility (*n* = 2033)		
	DOR + asthenozoospermia	217 (10.7)
	DOR + azoospermia	101 (4.9)
	DOR + oligozoospermia	596 (29.3)
	PMOS + asthenozoospermia	120 (5.9)
	PMOS + azoospermia	127 (6.2)
	PMOS + oligozoospermia	386 (18.8)
	Endometriosis/Endometrioma + asthenozoospermia	57 (2.8)
	Endometriosis/Endometrioma + oligozoospermia	12 (0.6)
	Endometriosis/Endometrioma + azoospermia	182 (9.0)
	Tubal factor + asthenozoospermia	65 (3.2)
	Tubal factor + oligozoospermia	4 (0.2)
	Tubal factor + azoospermia	150 (7.5)
	Anovulation + male factors	3 (0.8)
	Myoma + male factors	5 (1.4)
	Polyp + male factors	2 (0.6)
	Advanced maternal age + male factors	4 (1.1)

* The following conditions have been identified: oocyte cryopreservation, advanced maternal age, uterine myomas, anovulation, endometrial polyps, adenomyosis, cervical insufficiency, breast cancer, and vaginismus. PMOS: polyendocrine metabolic ovarian syndrome, DOR: diminished ovarian reserve.

**Table 4:** Comparison of female, male, combined, and unexplained infertility groups for frequency of occurrence, mean maternal and paternal ages, antral follicle count, the number of MII oocytes, pregnancy rates, and live birth rates in 7478 cases undergoing ET *.

		^1^ Female	^2^ Male	^3^ Combined	^4^ Unexplained	*p*	Post Hoc
Frequency of occurrence n (%)		2468 (33.00)	1999 (26.7)	1664 (22.2)	1347 (18.00)		
^a^ Maternal age, mean ± SD		35.20 ± 5.77	33.65 ± 5.40	34.75 ± 6.06	33.88 ± 4.85	0.000 ***	1, 3 > 2, 4
^a^ Paternal age, mean ± SD		37.22 ± 6.18	36.88 ± 6.27	37.81 ± 6.63	36.37 ± 5.39	0.000 ***	3 > 1, 2 3 > 4 1 > 4
^a^ Antral follicle count, mean ± SD		10.18 ± 8.37	10.37 ± 5.31	11.76 ± 9.21	12.16 ± 3.55	0.000 ***	3, 4 > 1, 2
^a^ M II oocyte number, mean ± SD		4.67 ± 4.05	5.34 ± 3.39	5.27 ± 4.60	6.22 ± 2.86	0.000 ***	4 > 2, 3 > 1
^b^ β-HCG positive cases %		41.49	47.67	37.86	46.77	0.001 **	2, 4 > 1, 3
Live birth in cases undergoing ET %		19.49	24.11	18.32	27.91	0.000 ***	4 > 2 > 1, 3
^b^ Pregnancy outcomes in β-HCG positive cases %	Live birth	46.97	50.57	48.34	59.68	0.000 ***	4 > 2 > 1, 3
	Ectopic (%)	5.30	2.90	5.10	2.40		1, 2, 3, 4
	Chemical (%)	26.70	24.40	28.40	19.80	0.008 **	1, 2, 3 2, 4 4 < 1, 3
	Abortion (%)	14.60	14.20	13.70	12.50		1, 2, 3, 4
	Not available (%)	6.40	7.90	4.40	5.60		1, 2, 3, 4

^a^ one-way ANOVA. Post-hoc pairwise comparisons were performed using Tukey. ^b^ Pearson’s chi-square test. Post-hoc pairwise comparisons were performed using the Bonferroni correction. ^1^ Female infertility; ^2^ Male infertility; ^3^ Combined infertility; ^4^ Unexplained infertility. Symbols (1, 2, 3 and 4) indicate the groups compared in the post hoc analysis. * *p* < 0.05, ** *p* < 0.01, *** *p* < 0.001.

## Data Availability

The datasets generated and/or analyzed during the current study are not publicly available due to patient privacy and institutional restrictions. However, they are available from the corresponding author, provided permission has been obtained from the institutional ethics committee.

## Abbreviations

The following abbreviations are used in this manuscript:

ART	Assisted Reproductive Technology
IVF	In Vitro Fertilization
ICSI	Intracytoplasmic Sperm Injection
ET	Embryo Transfer
FET	Frozen Embryo Transfer
BMI	Body Mass Index
FSH	Follicle-Stimulating Hormone
LH	Luteinizing Hormone
E2	Estradiol
AFC	Antral Follicle Count
MII	Metaphase II Oocyte
2PN	Two Pronuclei (Normal Fertilization)
β-hCG	Beta Human Chorionic Gonadotropin
LBR	Live Birth Rate
CPR	Clinical Pregnancy Rate
OPU	Oocyte Pick-Up
GnRH	Gonadotropin-Releasing Hormone
OHSS	Ovarian Hyperstimulation Syndrome
SD	Standard Deviation
SEM	Standard Error of the Mean
SPSS	Statistical Package for the Social Sciences
